# 3-year follow-up of a prospective, multicenter study of the Amplatzer Piccolo™ Occluder for transcatheter patent ductus arteriosus closure in children ≥ 700 grams

**DOI:** 10.1038/s41372-023-01741-1

**Published:** 2023-08-16

**Authors:** Brian H. Morray, Shyam K. Sathanandam, Thomas Forbes, Matthew Gillespie, Darren Berman, Aimee K. Armstrong, Shabana Shahanavaz, Thomas Jones, Toby Rockefeller, Henri Justino, David Nykanen, Courtney Weiler, Dan Gutfinger, Evan M. Zahn

**Affiliations:** 1 Division of Pediatric Cardiology, Seattle Children’s Hospital, Seattle, WA USA; 2 LeBonheur Children’s Hospital, Memphis, TN USA; 3 Joe DiMaggio Children’s Hospital, Hollywood, FL USA; 4 Children’s Hospital of Philadelphia, Philadelphia, PA USA; 5 Children’s Hospital of Los Angeles, Los Angeles, CA USA; 6 Nationwide Children’s Hospital, Columbus, OH USA; 7 Cincinnati Children’s Hospital, Cincinnati, OH USA; 8 Mercy Children’s Hospital, Kansas City, MO USA; 9 Rady Children’s Hospital, San Diego, CA USA; 10 Arnold Palmer Hospital, Orlando, FL USA; 11 Abbott Structural Heart, Santa Clara, CA USA; 12 Cedars-Sinai Medical Center, Los Angeles, CA USA

**Keywords:** Congenital heart defects, Outcomes research, Paediatrics

## Abstract

**Objective:**

This study describes 3-year follow-up of 200 infants weighing ≥ 700 grams who underwent transcatheter patent ductus arteriosus (PDA) closure with the Amplatzer Piccolo™ Occluder.

**Study Design:**

Between June 2017 and February 2019, 200 children were enrolled in this U.S. study (NCT03055858). PDA closure, survival, and device- or procedure-related events were evaluated. A total of 156 of the available 182 patients (86%) completed the study.

**Results:**

The implant success rate was 95.5% (191/200). At 3 years, PDA closure was observed in 100% (33/33) of patients. Survival was >95% with 9 reported deaths. No deaths were adjudicated as device- or procedure-related. Notable events included aortic obstruction (2) requiring stent placement and tricuspid regurgitation (5), for which no interventions were required.

**Conclusions:**

This follow-up study demonstrates high rates of PDA closure, low serious complication rates, and survival > 95% at 3 years. The Amplatzer Piccolo™ Occluder is a safe and effective therapy for PDA treatment in premature infants. ClinicalTrials.gov identifier: NCT0305585.

## Introduction

The presence of a hemodynamically significant patent ductus arteriosus (PDA) is common in low birth weight and extremely low birth weight premature infants and has been associated with an increased risk of necrotizing enterocolitis, chronic lung disease, intraventricular hemorrhage, and death [1]. Trials of medical or surgical treatment of the PDA have generally failed to demonstrate reductions in these adverse clinical outcomes, and there is continued debate on how best to treat infants with a hemodynamically significant PDA [1, 2]. Transcatheter PDA device closure, which is standard practice for the management of PDA in older children and adults, has recently emerged as a treatment option for the management of the PDA in preterm infants [3].

The Amplatzer Piccolo™ Occluder (Abbott Structural Heart, Plymouth, MN), previously called the Amplatzer™ Ductal Occluder II Additional Sizes (ADO II AS), received the CE Mark for PDA closure in infants ≥ 6 kg in 2011. Based on published literature [3, 4] demonstrating the safety and efficacy of transcatheter closure of the PDA in preterm infants < 6 kg, a pre-market trial of the Amplatzer Piccolo™ Occluder was conducted in the United States and enrolled 200 children for transcatheter device closure of the PDA [5]. The 6-month results demonstrated a high procedural success rate ( > 95%) and a low rate of major complications ( < 3%). Based on these data, the Amplatzer Piccolo™ Occluder received approval for commercial use in the United States from the Food and Drug Administration in 2019 for PDA closure in patients ≥ 700 grams. The procedural outcomes and 6-month clinical data were previously published [5]. Here, we report the 3-year clinical outcomes from this cohort of infants and children.

## Methods

The ADO II AS Clinical Study (ClinicalTrials.gov identifier: NCT03055858) was a single arm, prospective, multicenter, pre-market clinical investigation designed to characterize the safety and effectiveness of the Amplatzer Piccolo™ Occluder (Abbott, Plymouth, MN, USA) in children ≥ 700 g and ≥ 3 days of age at the time of implant. The study included an investigational device exemption (IDE) cohort with 50 patients enrolled between 05 June 2017 and 25 January 2018 at 8 clinical sites, and a continued access protocol (CAP) cohort with 150 patients enrolled between 26 March 2018 and 01 February 2019 at 9 sites. Both the IDE and CAP cohorts enrolled patients in the United States and followed the same study protocol. Each study site received approval from the local Institutional Review Board prior to any study conduct or patient enrollment. This study was performed in accordance with the Declaration of Helsinki.

Key inclusion criteria were the presence of a PDA ≤ 4 mm in diameter and ≥ 3 mm in length based on an intra-procedural echocardiogram or angiogram. Key exclusion criteria included weight < 700 g and age < 3 days at time of the procedure, presence of a pre-existing coarctation of the aorta or left pulmonary artery (LPA) stenosis, pulmonary hypertension with cardiac output dependent on a right to left shunt through the PDA, presence of an intracardiac thrombus, or active infection. Demographic information and common morbidities of prematurity were diagnosed prior to enrollment but collected on study case report forms once the patient was enrolled. Patient follow-up occurred prior to hospital discharge and at 30-days and 6-months post-procedure. Additional follow-up visits occurred at 1-, 2-, and 3-years post-implant. All IDE and CAP patients were assessed at each follow-up visit for adverse events.

The IDE cohort (*N* = 50) was required to undergo transthoracic echocardiography (TTE) at each follow-up visit. The study protocol did not require CAP patients (*N* = 150) to undergo TTE beyond 6-months post-procedure unless there was a persistent residual shunt observed at 6 months. PDA closure was defined as the presence of either no (Grade 0) or a trivial residual shunt (Grade 1), as assessed by an independent Echocardiography Core Laboratory (core lab) [5]. A trivial shunt was defined as a narrow color Doppler jet through or around the device that did not extend beyond the LPA. The core lab reviewed baseline (pre-implant) and 6-month TTEs for IDE and CAP patients, as well as annual TTEs for IDE patients. An elevated LPA or aortic peak Doppler velocity was defined as > 2.5 m/s by echocardiography at any time during follow-up [6]. Independent adjudication of pre-defined clinical events, including major and minor complications and deaths, was performed by an independent clinical events committee (CEC). A major complication was defined as a device- or procedure-related adverse event resulting in death, life-threatening adverse event, persistent or significant disability/incapacity, and/or a major open surgical intervention.

Procedural success was defined as having the Amplatzer Piccolo Occluder positioned within the PDA by the end of the implant procedure. Patients who did not receive the Amplatzer Piccolo Occluder or had the Amplatzer Piccolo Occluder removed were withdrawn from the study after 30 days of observation. The unique procedural techniques required for this population, as well as the primary outcomes of effectiveness (rate of effective closure at 6 months) and safety (rate of major complications through 180 days) were previously described in the 6-month follow-up publication [5]. Survival, LPA and aortic obstruction, and tricuspid regurgitation (TR) for the entire study cohort, as well as PDA closure throughout follow-up for the IDE cohort are described here. Findings at autopsy are also reported.

### Statistical methods

This is a single-arm descriptive study. Categorical data are reported as a count and percentage. Continuous data are reported as mean ± SD unless otherwise specified. The range is also provided to present the minimum and maximum values. Patient survival was analyzed using the Kaplan-Meier method. Subjects with a death or unknown survival status were censored on the last known date reported alive. Peak instantaneous Doppler gradients of the LPA and aorta were obtained as part of the study protocol and converted using the simplified Bernoulli equation to calculate peak velocities. Data were analyzed by using SAS software, version 9.4 (Chicago, IL).

## Results

During the IDE and CAP study periods, a total of 200 patients were enrolled. The entire study cohort was evenly split by procedural weight ≤ 2 kg (*n* = 100) and > 2 kg (*n* = 100). A total of 132 infants were referred for PDA closure from the neonatal intensive care unit (NICU; Table 1). The infants from the NICU had an average birth weight of 0.85 ± 0.39 kg and an average gestational age at birth of 26 ± 3 weeks. Of the NICU infants, 91% were on mechanical respiratory support and 7% were on inotropic support at the time of PDA closure. Comorbidities of prematurity in the infants from the NICU included 78% with respiratory distress syndrome, 41% with a history of an intraventricular hemorrhage, 21% with a history of sepsis, and 14% with a history of necrotizing enterocolitis. Prior treatment with a cyclooxygenase (COX) inhibitor was performed in 37% of the infants from the NICU. Medical management prior to Piccolo implant was at the discretion of the institution. Of the 9 study sites, 7 had low usage ( < 30%) of COX inhibitors prior to the procedure.

**Table 1:** Demographics and Comorbidities Prior to Implant.

Characteristics	≤ 2 kg (*N* = 100)	> 2 kg (*N* = 100)	Total (*N* = 200)
Age at procedure, months	1.25 ± 0.6 [0.3, 3.15]	26.58 ± 44.32 [0.49, 216.80]	3.92 ± 33.74 [0.30, 216.80]
Sex, male	60 (60.0%)	42 (42.0%)	102 (51.0%)
Procedure weight (kg)	1.25 ± 0.35 [0.70, 2.00]	11.25 ± 13.52 [2.02, 68.50]	6.25 ± 10.77 [0.70, 68.50]
Referred from NICU^a^	100 (100.0%)	32 (32.0%)	132 (66.0%)
Birth Weight (kg)	0.75 ± 0.20 [0.43, 1.50]	1.14 ± 0.63 [0.50, 3.04]	0.85 ± 0.39 [0.43, 3.04]
Gestational Age at Birth	25.3 ± 1.9 [22,34]	28.2 ± 4.4 [22,42]	26.0 ± 3.0 [22,42]
Procedure weight (kg)	1.25 ± 0.35 [0.70, 2.00]	2.86 ± 0.91 [2.02, 5.50]	1.64 ± 0.88 [0.70, 5.50]
RDS	87% (87/100)	50% (16/32)	78% (103/132)
IVH	42% (42/100)	38% (12/32)	41% (54/132)
Sepsis	25% (25/100)	9% (3/32)	21% (28/132)
ROP	17% (17/100)	34% (11/32)	21% (28/132)
NEC	15% (14/100)	9% (3/32)	14% (18/132)
Mechanical Respiratory Support	96% (92/96)	70% (14/20)	91% (106/116)
Inotropic Support	7% (7/96)	5% (1/20)	7% (8/116)
Previous Treatment with COX Inhibitors	45% (45/100)	13% (4/32)	37% (49/132)

^a^Data presented corresponds to cohort of patients from the neonatal intensive care unit (NICU). *RDS* Respiratory Distress Syndrome, *IVH* Intra-Ventricular Hemorrhage, *ROP* Retinopathy of Prematurity, *NEC* Necrotizing Enterocolitis.

### Procedural outcomes

The acute procedural outcomes have been previously reported [5]. The acute procedural implant success rate was 95.5% (191/200), 92% in patients > 2 kg and 99% in patients ≤ 2 kg. There were nine unsuccessful implants including two cases of intraprocedural device embolization in which the PDA could not ultimately be closed and seven cases due to inability to achieve a stable device position (Supplementary Table 1). Of the 191 patients who underwent successful implant, 186 were still enrolled in the study at 6 months post-implant. A total of 180 patients completed the 6-month follow-up visit with an echocardiogram. Out of the 180 patients who completed the 6-month echocardiogram, 7 echocardiograms could not be read by the core lab, leaving a total of 173 patients with sufficient data to complete the 6-month echo-derived endpoint. Effective closure of the PDA by echocardiographic criteria was achieved in 99.4% of patients with available echocardiograms (172/173) at 6 months post-implant.

### 3-Year follow-up

**Fig. 1:**
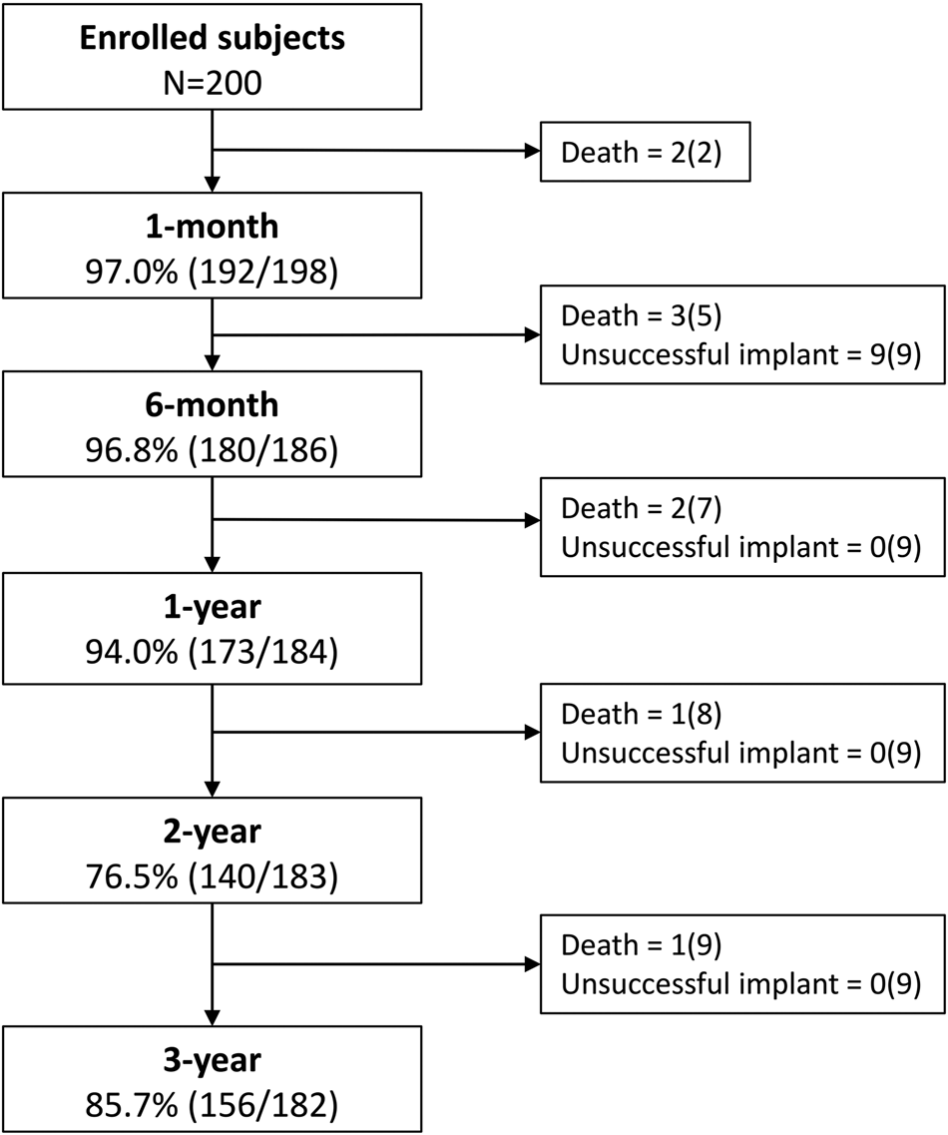
Flow chart of study enrollment and follow-up for both the IDE and CAP cohort through the three-year study period. A total of 182 patients were available for study follow-up of which 156 (85.7%) completed three-year follow-up.

**Fig. 2:**
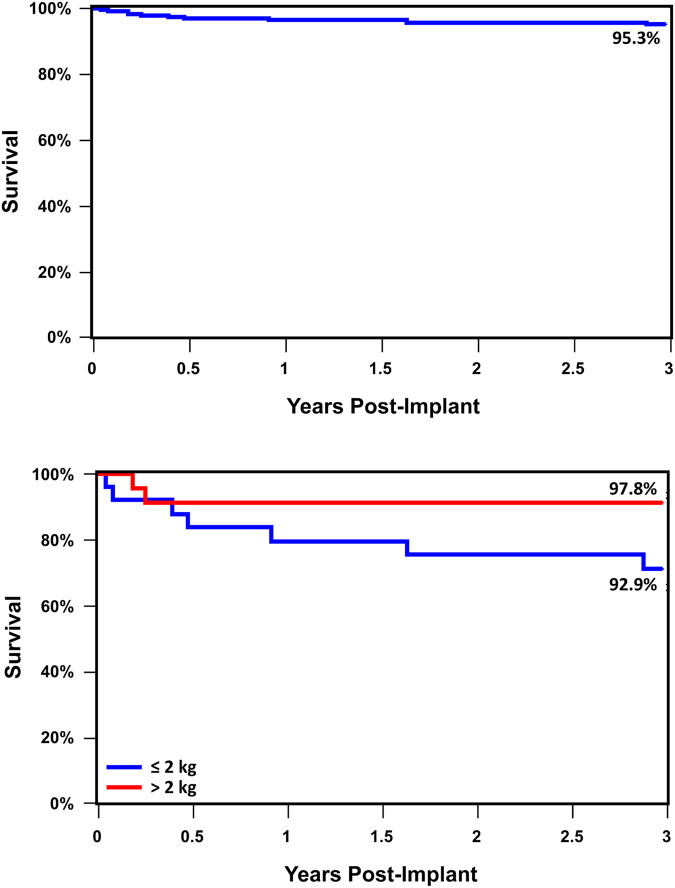
Three year study cohort survival curves. Overall survival for the entire cohort was 95% at three years. Kaplan-Meier survival analysis demonstrated no significant difference in three-year survival between patients >2 kg and ≤ 2 kg.

**Fig. 3:**
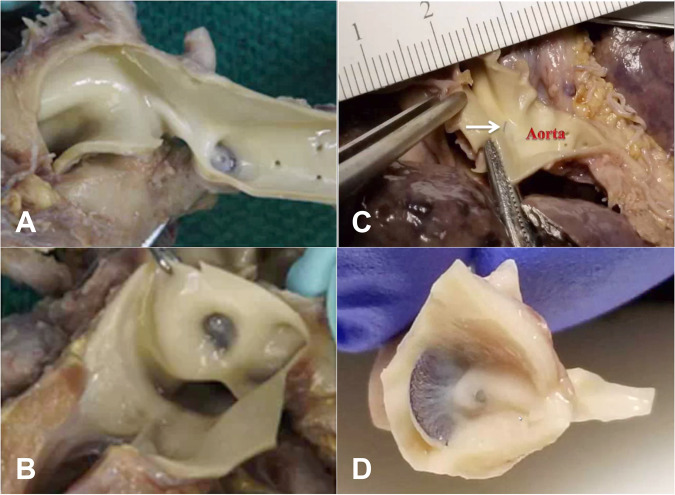
Postmortem histopathology following PDA occlusion with the Amplatzer Piccolo™ Occluder. **A**, **B** Term infant with a history of congenital heart block and respiratory failure died 66 days post-implant (Patient #9). Aortic (**A**) and Pulmonary Artery (**B**) views demonstrate complete endothelialization and intraductal device placement. **C**, **D** Preterm infant with a history of coarctation requiring stent placement and respiratory failure died 141 days post-implant (Patient #3). Aortic (**C**) and Pulmonary Artery (**D**) views demonstrate complete endothelialization and intraductal device placement.

Of the 200 patients, 9 were withdrawn from the study due to an unsuccessful implant. There were five patient deaths prior to the 6-month follow-up, making 186 patients eligible for the 6-month follow-up evaluation as described above. There were four additional patient deaths after six months, leaving 182 patients eligible for the 3-year follow-up (Fig. 1). Overall patient survival at 3 years was 95.3%, and 92.9% in children ≤ 2 kg (Fig. 2). Beyond 6-months there were no additional device or procedure related complications reported. All patient deaths were reviewed to determine relationship to the procedure and device (Supplementary Table 2). Of the 9 patients who died following device implant, 7 were ≤ 2 kg at the time of implant. Causes of death included respiratory failure (*n* = 6), bleeding following an unrelated abdominal surgery (*n* = 1), necrotizing enterocolitis (*n* = 1), and cerebral infarction (*n* = 1). The median time from device implant to death was 141 days (range 14–1046 days). After an independent review by the CEC, no deaths were adjudicated as device or procedure related. Among the 9 deaths, there were three patients who underwent an autopsy at 66 days (Patient #9), 141 days (Patient #3), and 593 days (Patient #1) post-implant. Autopsies demonstrated complete coverage of the device with neoendothelium (Fig. 3).

**Fig. 4:**
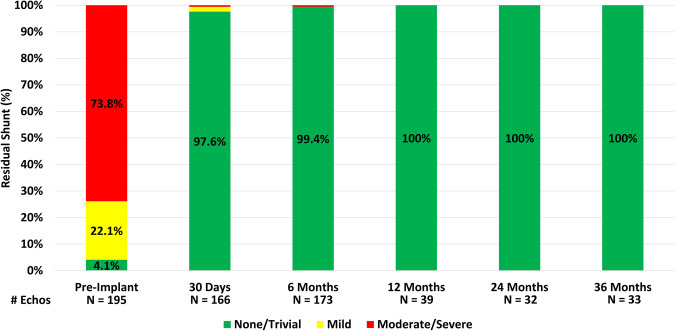
All patients with echocardiographic follow-up through three years demonstrated complete ductal occlusion with no residual shunt. At six-month follow-up there was one patient with a significant residual shunt.

Of the 50 patients enrolled as part of the IDE cohort, who were required to have imaging completed at each visit, 33 had echocardiograms available for review at the final 3-year follow-up. The findings demonstrated effective PDA closure in all patients (33/33) with echocardiograms (Fig. 4). Echocardiography follow-up demonstrated that effective PDA closure was achieved in 97.4% of patients 30-days post implant, increased to 99.4% at 6-months and was subsequently sustained at 100% through 3-years of follow-up for the IDE cohort. There were no cases in which the PDA reopened following documented closure.

### Adverse events

Intraprocedural device embolization occurred in 5 patients (2 patients ≤ 2 kg). Three cases were successfully closed with larger Piccolo devices, while two cases could not be closed. Post procedure migration occurred in 2 additional patients (1 patient ≤ 2 kg). All cases of device embolization and migration were successfully managed by transcatheter device retrieval using a snare. Major device or procedure related complications through 6 months occurred in 4 patients (2 transfusions, 1 hemolysis, 1 aortic obstruction).

Post procedure aortic obstruction requiring intervention with stent placement occurred in 2 patients ≤ 2 kg. One of the cases with mild aortic coarctation prior to the procedure was adjudicated by the CEC as not related to the device or the implant procedure. The coarctation was relieved with stent placement and the patient underwent elective balloon dilatation to 5 mm at 3 months post-implant followed by surgical coarctation repair at 16 months of age. At the time of the surgical coarctation repair (476 days post-implant) it was noted that the device was completely covered with a neoendothelium. Once the coarctation was surgically repaired, the aortic velocities were no longer elevated. The second instance of aortic obstruction was adjudicated as device- and procedure-related. That patient died 8 days following stent placement secondary to severe hypoxemic and hypercarbic respiratory failure (Supplementary Table 2; Patient #2). At the 6-month follow-up there was a third patient with an elevated aortic velocity >2.5 m/sec which resolved spontaneously with somatic growth. At 6-month follow-up an elevated LPA velocity > 2.5 m/sec was observed in 2 patients. Neither patient required intervention and the elevated LPA velocities decreased during the follow-up period. One of these patients subsequently died of a cerebral infarct **(**Supplementary Table 2; Patient #8).

An increase in TR was reported in 5 patients (all < 2 kg) following the implant procedure. Two cases of severe TR occurred following retrieval of an embolized device, two cases with moderate TR were due to an unknown cause, and one case of mild TR was associated with difficulty advancing a catheter through the heart. Of the 5 patients, 1 with moderate TR died prior to the 6-month follow-up from necrotizing enterocolitis (Patient #7) and 1 infant with severe TR who had a post-implant device migration was withdrawn from the study following transcatheter device retrieval as a subsequent device was not placed. In the three remaining patients, one had mild TR at the most recent follow-up and the two remaining patients continued to have moderate and severe TR at 3-years post-implant that was unchanged from prior echocardiograms. The patient with severe, persistent TR had a flail septal leaflet. It is unclear if the tricuspid valve injury was sustained during initial device implant or during the extraction of the embolized device. At the 3-year follow-up no patients required an intervention to repair or replace the tricuspid valve.

## Discussion

Clinical outcomes from the IDE and CAP cohorts demonstrate continued safety with low rates of serious complications and high rates of effective transcatheter PDA closure with the Amplatzer Piccolo™ Occluder in pediatric patients through 3-years of follow-up. Survival was > 95% out to 3 years with no deaths attributed to the procedure or device. Successful device implant was achieved in > 95% of patients and 99% of patients ≤ 2 kg. While only the IDE cohort was required to undergo echocardiography through 3 years of follow-up, all IDE patients with available echocardiograms (*n* = 33) demonstrated complete occlusion of the PDA at 3 years and there were no reported instances of the ductus arteriosus reopening. Major complications were reported in 2.1% of cases. Intraprocedural device embolization (2.5%) and increased TR (2.5%) were the most commonly observed adverse events during the 6-month follow-up. Beyond 6-months there were no additional device or procedure related complications reported. None of the three patients with increased TR followed through study completion required an intervention on the valve. Clinically significant aortic obstruction occurred in two patients in the first week post-procedure, requiring stent placement to relieve the obstruction in both cases. There were no late cases of aortic obstruction and no cases of LPA obstruction requiring intervention through 3-years of follow-up. Great attention should be taken to assess the device prior to release to minimize the risk of LPA or aortic obstruction.

Other contemporary studies of the Amplatzer Piccolo™ Occluder have demonstrated similar rates of successful closure and adverse events [6–12]. One study, however, documented a high rate of LPA obstruction and device deformation (i.e., lengthening) in a small sample of infants < 2.5 kg (*N* = 10) undergoing PDA closure [13]. This may have been due to a different approach to device sizing than used in this IDE/CAP study, and the devices deployed in the cases with LPA obstruction were generally larger (both in diameter and length) than what would have been deployed in our study. A meta-analysis of 373 transcatheter PDA closures performed with a variety of devices in infants < 1.5 kg from 28 studies documented a technical success rate of 96% and a serious adverse event rate of 8% [14]. Multivariate analysis demonstrated younger age at implant to be a risk factor for serious adverse events. Technical success increased according to the year of publication, and the frequency of adverse events was higher in lower volume centers. The results suggest that while transcatheter PDA closure is generally a safe and effective procedure, this is a fragile population of patients and procedural experience is important to maximize technical success and reduce the rate of adverse events. Techniques for preventing and managing periprocedural complications in this population have been published and serve as useful guidelines for institutions building a program for transcatheter PDA closure in preterm infants [7].

The development of a device specific for PDA closure in preterm infants coincided with new procedural techniques to reduce the risk of vascular and device related complications. Most importantly, this procedure is performed with ultrasound guided venous access only and a greater emphasis on complete intraductal placement of the device in infants ≤ 2 kg to minimize protrusion of the device into the aorta or the LPA compared to larger children ( > 2 kg) where the device size is selected so that the central waist spans the entire length of the ductus with the retention discs placed just outside the ductus to improve positional stability and minimize the potential for device embolization. This approach requires navigation through the cardiac structures in a small, preterm infant and necessitates increased dependence on intraprocedural transthoracic echocardiography to assist with proper device placement. As experience has grown, so too has the understanding about the subtleties of device sizing and device positioning within the ductus to minimize the risk of device embolization and malposition. An increase in TR occurred in 5% of infants ≤ 2 kg (5/100) in the trial. Proposed mechanisms for TR include delivery catheter and wire mismatch, advancement of a catheter underneath chordae, prolonged cases with embolization or malposition necessitating device retrieval, or the stiffness of the delivery catheter and wire causing damage to the valve. Based on some of these proposed mechanisms, guidelines for prevention have been proposed, [7] and new techniques for crossing the tricuspid valve have been proposed with early results showing a promising reduction in the rates of tricuspid valve injury [15]. Additional on-going efforts to modify the delivery system may help to reduce the rate of tricuspid valve injury further.

While this current study was designed to demonstrate the safety and effectiveness of the Amplatzer Piccolo Occluder in treating the PDA in pediatric patients, there is still much work to do in defining what impact this procedure will have on the clinical course of these patients. Defining the appropriate target population with a hemodynamically significant PDA and time window for intervention will enhance the ability to apply these techniques to the right patients. There remains a lack of uniformity in how a hemodynamically significant PDA is defined, which has significantly hampered the outcomes of prior trials of PDA management. A PDA severity score has been developed and demonstrated to predict chronic lung disease or death before discharge in an observational study of infants born < 29 weeks gestational age [16]. Future clinical studies of transcatheter PDA closure will need to rely on validated scoring systems to target patient populations with hemodynamically significant PDAs. Part of the determination of hemodynamic significance is the duration of exposure to a large left to right shunt. Some early work has already been done demonstrating that earlier PDA closure before 4 weeks of age reduces the hemodynamic impact of the left to right shunt on the pulmonary vasculature, improves respiratory severity scores, reduces duration of mechanical ventilation, and decreases time to discharge [12, 17].

Ultimately, a randomized controlled trial of transcatheter device closure compared with conventional medical therapy is necessary to establish the clinical benefit of transcatheter closure. The Percutaneous Intervention Versus Observational Trial of Arterial Ductus in Low Weight Infants (PIVOTAL) trial (ClinicalTrials.gov Identifier: NCT05547165) has been approved and, beginning in 2023, will randomize mechanically ventilated patients between 7 and 32 days of age with a hemodynamically significant PDA to transcatheter PDA device closure or responsive medical management of the PDA. This represents an important step in establishing which patients will derive clinical benefit from this procedure.

As the techniques for transcatheter closure improve and the target patient population is refined, the next frontier is bringing this procedure closer to the patients it serves. Transporting these fragile patients is not without risk, and some experienced centers are endeavoring, in a stepwise fashion, to bring this procedure out of the catheterization lab and directly to the bedside in the NICU [18, 19]. This offers the benefit of allowing experienced practitioners to continue to care for these patients in the units that know them best without the need for transport. In the future, bedside closure may allow experienced operators to bring this procedure to locations and NICUs that do not traditionally have access to the expertise of a pediatric cardiac center [20].

### Limitations

This study was designed to study the safety and effectiveness of transcatheter PDA closure using the Amplatzer Piccolo™ Occluder in pediatric patients. It was not designed to determine whether transcatheter closure offers clinical benefit compared to medical management or conservative therapy. Additionally, only the IDE cohort was followed with serial echocardiography through 3 years of follow-up. The CAP cohort underwent echocardiographic follow-up through 6 months and clinical follow-up for adverse event and mortality tracking through 3 years.

## Conclusions

Transcatheter closure of the PDA in pediatric patients with the Amplatzer Piccolo™ Occluder is safe and effective with > 99% procedural success in patients ≤ 2 kg, 100% closure documented out to 3-years in the IDE cohort, and overall cohort survival > 95% at 3-years. Major procedure or device related complications were uncommon. These data are an important steppingstone to improving procedural techniques and safety, establishing clinical benefit of transcatheter PDA closure in at-risk preterm infants and extending this procedure beyond the cardiac catheterization lab to the bedside. The availability of a safe and effective intervention for the PDA in preterm infants will help explore important questions about the impact of the PDA on the management of preterm infants and have a significant impact on clinical outcomes for this at-risk population.

### Supplementary information

Supplemental data

## Data Availability

The data that support the findings of this study are not openly available but may be available from the corresponding author upon reasonable request. Data are located in controlled access data storage at Abbott.
