# Supplementary material for: 3-year follow-up of a prospective, multicenter study of the Amplatzer Piccolo™ Occluder for transcatheter patent ductus arteriosus closure in children ≥ 700 grams
Source: J Perinatol. 2023 Aug 16;43(10):1238–44. doi: 10.1038/s41372-023-01741-1 (PMC10541325; doi:10.1038/s41372-023-01741-1)
Supplement: Supplementary file 1 — Supplemental data [file 41372_2023_1741_MOESM1_ESM.docx]

**Supplemental Table 1: Unsuccessful Implant Listing**

| **Subject ID** | **Procedure Weight (kg)** | **PDA Type** | **Minimum PDA Diameter by Angiography (mm)** | **Maximum PDA Diameter by Angiography (mm)** | **PDA Length by Angiography (mm)** | **Device Size Attempted** | **Reason for Unsuccessful Implant** | **Subsequent PDA Closure** |
| --- | --- | --- | --- | --- | --- | --- | --- | --- |
| 10 | 4.6 | D | 3.60 | 5.40 | 7 | 05-04 | Intra-procedural device embolization | Other Amplatzer™ product |
| 11 | 6 | A | 3.10 | 5.80 | 17 | 05-04 | Intra-procedural device embolization | Other Amplatzer product |
| 12 | 1.6 | B | 3.20 | 3.40 | 5.30 | 05-02 | Unable to achieve stable position | Surgical ligation |
| 13 | 2.3 | UNK | 3 | 4.20 | 10.50 | 05-04 05-02 | Unable to achieve stable position | Surgical ligation |
| 14 | 2.6 | B | 3 | 6.20 | 6.10 | 05-02 05-04 05-06 | Unable to achieve stable position | Surgical ligation |
| 15 | 4.3 | A | 3.70 | 4.20 | 5.20 | 05-02 | Unable to achieve stable position | Closure postponed |
| 16 | 5.5 | E | 3.10 | 6 | 13.60 | 05-06 | Unable to achieve stable position | Other Amplatzer product |
| 17 | 5.5 | F | 3.70 | 5.30 | 13.50 | 05-06 05-04 | Unable to achieve stable position | Other Amplatzer product |
| 18 | 11.5 | A | 3.60 | 3.80 | 9.70 | 05-06 | Unable to achieve stable position | Other Amplatzer product |

**Supplementary Table 2: Mortality Listing**

| **Subject ID** | **Gestational Age at Birth** | **Birth Weight** | **Procedure Weight** | **Time Between Procedure and Death (days)** | **Age at Death (days)** | **Medical History** | **Cause of Death** | **Autopsy** |
| --- | --- | --- | --- | --- | --- | --- | --- | --- |
| 1 | 22 weeks | 500g | 800g | 593 | 640 | RDS, IVH, NEC, bowel resection | Intra-abdominal bleeding – following exploratory laparotomy. | Yes |
| 2 | 24 weeks | 580g | 800g | 14 | 48 | RDS, IVH, BPD, PFO | Respiratory failure - PHTN leading to cardiorespiratory arrest. Developed aortic coarctation due to device aortic obstruction, drug eluting stent placed. Continued to have RDS and maintained on HFV. Continued to have severe acidosis and carbon dioxide retention. | No |
| 3 | 24 weeks | 510g | 1020g | 141 | 194 | RDS, sepsis, chronic lung disease, BPD, PHTN | Respiratory failure - secondary to pneumonia. | Yes |
| 4 | 25 weeks | 681g | 1200g | 1046 | 1088 | Duodenal atresia | Accidental tracheostomy decannulation. | Not Reported |
| 5 | 25 weeks | 495g | 1700g | 171 | 251 | RDS, retinopathy, IVH, sepsis, BPD, PHTN, IUGR, maternal HELLP, opioid dependence | Respiratory failure - complications of prematurity and lung disease. | Not reported |
| 6 | 26 weeks | 590g | 2300g | 91 | 176 | IVH, NEC, respiratory failure, acute hypoxemic respiratory failure, hypoglycemia, anemia, thrombocytopenia, BPD | Respiratory failure - Due to decreased liver function and need for respiratory support, family decided to extubate. | Not Reported |
| 7 | 26 weeks | 584g | 900g | 14 | 48 | RDS, IVH, Cholestatic jaundice, maternal HELLP syndrome | NEC | No |
| 8 | 28 weeks | 1060g | 1000g | 333 | 343 | PFO, supravalvular aortic narrowing, tricuspid regurgitation, pulmonary valve stenosis, mild mitral stenosis, hypoplastic aortic valve | Cerebral infarcts - Family decided on palliative care, subject was extubated | No |
| 9 | Term | N/A | 3400g | 66 | 81 | Temporary pacemaker, complete heart block secondary to maternal SLE/Sjogren’s antibodies. | Respiratory failure - secondary to PHTN, cardiac insufficiency, pneumonia. | Yes |

RDS = respiratory distress syndrome, IVH = intraventricular hemorrhage, BPD = bronchopulmonary dysplasia, PFO = patent foramen ovale, PHTN = pulmonary hypertension, NEC = necrotizing enterocolitis, HELLP = hemolysis, elevated liver enzymes, and low platelet counts, IUGR = intrauterine growth restriction, SLE = systemic lupus erythematosus.
